# Krait Snake Bite Presenting as a Cerebral Salt Wasting

**DOI:** 10.5005/jp-journals-10071-23214

**Published:** 2019-07

**Authors:** Niraj Kumar Keyal, Raju Shrestha, Sumal Thapa, Pooja Adhikari

**Affiliations:** 1 Department of Critical Care and Emergency Medicine, B & C Medical College Teaching Hospital and Research Centre, Birtamode, Jhapa, Nepal; 2-4 Department of Anaesthesia, B & C Medical College Teaching Hospital and Research Centre, Birtamode, Jhapa, Nepal

## Abstract

**How to cite this article:** Keyal NK, Shrestha R, Thapa S, Adhikari P. Krait Snake Bite Presenting as a Cerebral Salt Wasting. Indian J Crit Care Med 2019;23(7):347–348.

Sir,

Snakebite is a serious public health problem in most of the south Asian countries including Nepal.^1^ Snake venoms contain more than 20 different constituents. Clinical feature are categorized into hematotoxic, neurotoxic and myotoxic. Postsynaptic neurotoxins bind to acetylcholine receptors at the motor endplate. Presynaptic neurotoxins release acetylcholine at neuromuscular junction, preventing further release of transmitter.^2^ This leads to ptosis, ophthalmoplegia and paralysis of respiratory muscles. Here, we report a case of krait snakebite that developed polyuria, hyponatremia and cerebral salt wasting.

A 16-years-old female weighing 50 kg, presented with history of loss of consciousness, drooping of eyelids (Fig. 1), and vomiting after krait snake bite. At presentation her Glasgow Coma Scale (GCS) was 7/15. Vital signs was within normal limit except respiratory rate which was 8 breaths/minute. On auscultation chest bilateral crepitation was present. Immediately the patient was intubated. At the time of intubation, the patient was able to move her both upper limbs. Two hours after intubation, pupils were bilateral dilated and non-reactive, corneal, plantar, and gag reflex were absent but was able to move both feet on command. She had already received 15 vial of polyvalent anti-snake venom (ASV) before referring to our hospital. Five vials of ASV was repeated and inj neostigmine 0.05 mg/kg with inj glycopyrrolate 0.01 mg/kg were given three doses. There was no improvement in ptosis so, 10 vials of ASV was repeated after 6 hours. All the routine laboratory parameters were within normal limits. On admission, her serum sodium level was 139 mEq/L with urine output of 50 mL/hour, hematocrit 30% and serum albumin 3.4 gm/dL but on day 2nd her plasma sodium dropped to 130 mEq/L, urine output of 200–300 mL/hour, hematocrit 35% and serum albumin 3.9 gm/dL. Thereafter, plasma sodium was ordered daily which revealed plasma sodium 125 mEq/L. On day 3rd, urine output of 200–300 mL/hour, hematocrit 39% and serum albumin 4.1 gm/dL. Urinary sodium was 262.5 mmol/L, urine osmolality was 671 mOsm/kg of water and urine specific gravity was 1.010. Three percent sodium chloride was started. She started to move her both lower limbs and upper limbs on 4th day in response to command (Fig. 2). She regained her consciousness, corneal, gag and plantar reflex on 5th day. The patient was extubated on 5th day of admission and shifted to ward then discharged on day 13.

Snakebite is a common cause of mortality and morbidity in Nepal.^1^ Various studies have shown that Krait is a common neurotoxic snake in Nepal followed by cobra.^2,3^ Neuroparalytic snake bite is a common emergency situation encountered in tropical countries, and severe envenomation may mimic coma and brain death. The common neurological manifestations are alteration in level of consciousness, paresthesia, abnormalities of taste and smell, ptosis, ophthalmoplegia, limb weakness, respiratory failure, palatal weakness, difficulty in swallowing, neck muscle weakness, generalized flaccid paralysis and delayed sensory, neuropathy, respiratory paralysis and most of the neurological symptoms are noticed.^4^ Krait snake bite can also presents as hyponatremia with high sodium excretion in urine and cerebral salt wasting,^5^ which was present in our patient. The mechanism of action of hyponatremia is unknown. It is postulated that neurotoxins damage the brain and releases natriuretic protein that act on kidney to excrete sodium. In addition cerebral injury may increase sympathetic nervous system activity, elevating renal perfusion pressure and releasing dopamine. To conclude, krait snakebite can develop hyponatremia and cerebral salt wasting which was evidence by raised in hematocrit and albumin level. So, it should be evaluated and managed early as this can affect the patient outcome. The understanding of its pathophysiology, though, is still unclear and needs further investigations.

**Fig. 1:**
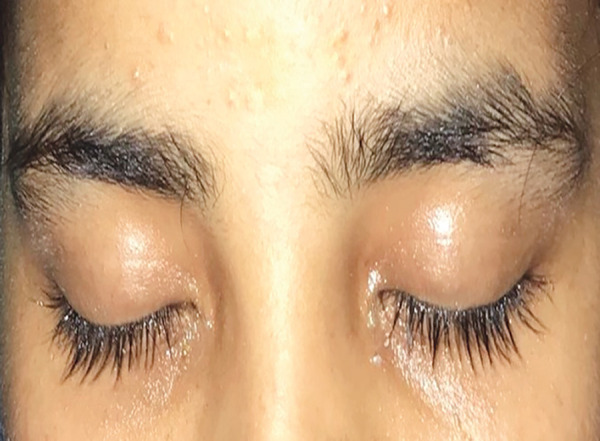
Patient before treatment

**Fig. 2:**
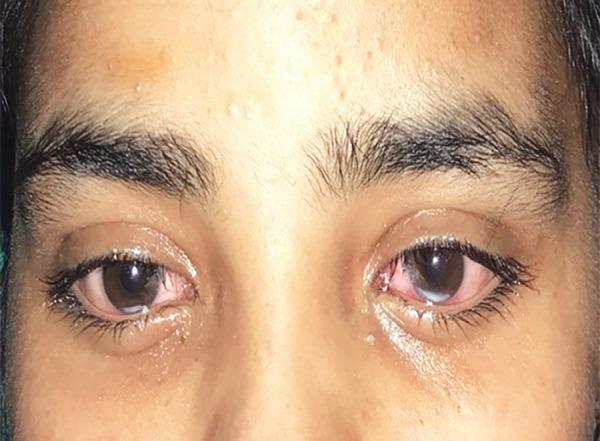
Patient after treatment
